# Impaired dynamic cerebral autoregulation: A potential mechanism of orthostatic hypotension and dementia in Parkinson’s disease

**DOI:** 10.3389/fnagi.2022.927009

**Published:** 2022-09-08

**Authors:** Hongxiu Chen, Erhe Xu, Fubo Zhou, Qiuping Li, Jingrong Zeng, Shanshan Mei, Yingqi Xing

**Affiliations:** ^1^Department of Vascular Ultrasonography, Xuanwu Hospital, Capital Medical University, Beijing, China; ^2^Beijing Diagnostic Center of Vascular Ultrasound, Beijing, China; ^3^Center of Vascular Ultrasonography, Beijing Institute of Brain Disorders, Collaborative Innovation Center for Brain Disorders, Capital Medical University, Beijing, China; ^4^Department of Neurology, Xuanwu Hospital, Capital Medical University, Beijing, China

**Keywords:** Parkinson’s disease, orthostatic hypotension, cognitive impairment, cerebral autoregulation, dementia

## Abstract

**Background:**

Orthostatic hypotension (OH) and cognitive impairment are common non-motor symptoms of Parkinson’s disease (PD). This study aimed to investigate whether impaired dynamic cerebral autoregulation (dCA) is associated with OH and Parkinson’s disease dementia (PDD), and analyze the related risk factors in patients with PDD.

**Materials and methods:**

We enrolled 89 patients with PD and 20 age- and sex-matched healthy controls (HCs). Cognition and different cognitive domains were assessed by the Montreal Cognitive Assessment scale. Non-invasive continuous beat-to-beat blood pressure and cerebral blood flow velocity were assessed using a servo-controlled finger plethysmograph and transcranial Doppler, respectively. dCA was examined using supine and orthostatic changes with transfer function analysis to derive the autoregulatory parameters of phase, gain, and coherence. Logistic regression analysis was performed to determine the risk factors for PDD.

**Results:**

We found that 21 (23.6%) patients with PD had OH. These patients showed worse cognitive performance in specific cognitive tasks, such as language and orientation. The patients with OH also had poorer dCA; the very low frequency (VLF) phase in two different postures was lower than that in patients without OH as well as HCs (both *P* < 0.05). And the normalized gain in the VLF and low frequency (LF) in standing position was higher in PD patients with and without OH than in HCs. PDD patients also had significantly higher LF normalized gain when standing than patients without dementia (*P* = 0.015), indicating impaired dCA. LF normalized gain in standing (odds ratio: 3.756, 95% confidence interval: 1.241–11.367) and education were significantly associated with PDD.

**Conclusion:**

Diminished dCA may represent a potential mechanism for OH and cognitive impairment and low educational level might be a significant factor contributing to the increased risk of PDD.

## Introduction

Parkinson’s disease (PD) is a neurodegenerative movement disorder clinically characterized by tremors, bradykinesia, and rigidity; it is also, however, associated with a wide range of non-motor symptoms (NMS) (Sveinbjornsdottir, 2016). Owing to improved management of motor symptoms in recent decades, numerous studies have found that NMS, such as cognitive impairment, autonomic dysfunction, sleep, and neuropsychiatric disturbances, typically emerge later in PD and generally increase in severity, thus affecting the quality of life of patients (Sveinbjornsdottir, 2016; Udow et al., 2016).

As a key symptom reflecting pure autonomic failure, orthostatic hypotension (OH) is a common non-motor manifestation of PD; recent studies have shown that its prevalence in PD is approximately 18–30% (Velseboer et al., 2011; Udow et al., 2016; Freeman et al., 2018). OH usually occurs after blood volume redistribution while standing, resulting in reduced cardiac output and cerebral hypoperfusion, thus manifesting with temporary symptoms, such as dizziness, blurred vision, falls, and even syncope (Freeman et al., 2011). Of note, a prospective cohort study revealed that OH is associated with a shorter survival in PD, and the 10-year survival rate in PD patients without OH was 93%, compared with only 74% in PD patients with OH (Goldstein et al., 2015).

Cognitive impairment is another cardinal NMS feature of PD; approximately 10% of patients with PD develop dementia every year, with an increased risk of four- to six-fold compared to healthy older people (Aarsland and Kurz, 2010). And a systematic review demonstrated that the point prevalence of Parkinson’s disease dementia (PDD) increases with disease duration (Aarsland et al., 2005). A multicenter 20-year study showed a cumulative prevalence rate of PDD of up to 83% in the second decade of the illness (Hely et al., 2008). Although they may appear as discrete clinical symptoms, a growing number of studies have shown a close relationship between cognitive impairment and OH in PD (Allcock et al., 2006; Udow et al., 2016; Longardner et al., 2020; Guo et al., 2021).

Recently, PD patients with OH were revealed to have a worse cognitive function in both cross-sectional and longitudinal analyses, with a mean follow-up of 5.3 years (Longardner et al., 2020). Among the 49 patients with PD and age- and sex-matched HCs, Tsai et al. (2009) evaluated the changes in systemic and cerebral hemodynamic responses to the cold pressor test and revealed that autonomic nervous system (ANS)-mediated cerebrovascular reactivity is impaired in PD. Moreover, another proof-of-concept study of 15 patients with Lewy body disorders [including 3 patients with PD-mild cognitive impairment (PD-MCI) and 5 with PDD] further showed that regional cerebral hypoperfusion was positively correlated with the severity of OH and may be implicated in domain-specific cognitive deficits (Robertson et al., 2016). Udow et al. (2016) demonstrated investigating functional neuroimaging of cerebral vascular autoregulation may help elucidate the putative pathophysiological link between OH and cognitive impairment. Based on the above, studying this potential mechanism of OH and cognitive impairment may promising to reduce the risk of PD with cognitive dysfunction through early diagnosis and effective intervention of OH. However, limited evidence is available on whether the potential dynamic cerebral autoregulation (dCA) between OH and dementia is impaired in patients with PD.

To elucidate the underlying mechanism between OH and cognitive deficits, we hypothesized that OH may be related to impaired dCA, resulting in cerebral hypoperfusion and further exacerbating cognitive impairment in PD. Thus, this study aimed to explore the influencing factors for PDD, assess the cognitive domain and dCA in PD patients with or without OH, and investigate whether dCA may be a potential mediator of OH and PDD.

## Materials and methods

### Study design and participants

Patients were consecutively recruited from the PD unit of Neurology of the Capital Medical University Xuanwu Hospital (Beijing, China) from June 2021 to December 2021. According to the UK Brain Bank diagnostic criteria, each patient with a clinically confirmed or probable diagnosis of PD was enrolled and diagnosed by two different neurologists (Hughes et al., 1992). Exclusion criteria were as follows: (1) poor or closed temporal window penetration; (2) atrial fibrillation or severe heart valve disease; (3) other diseases affecting dysautonomia and dCA, such as diabetes, severe intracranial or extracranial artery stenosis and occlusion, and stroke; and (4) other diseases affecting cognition or history of chronic psychiatric diseases, including major depressive and bipolar disorder. A total of 20 sex- and age-matched volunteers without a history of PD were recruited as healthy controls (HCs) from the same hospital.

This cross-sectional study was approved by the Ethics Committee of the Capital Medical University Xuanwu Hospital [approval number (2021)109], and all participants or their legal guardians provided written informed consent to participate in this study.

### Clinical assessments

We collected the following data on all study participants: (1) basic demographic data, such as age, gender, education, body mass index (BMI), and history of hypertension; (2) PD-related symptoms and severity, mainly including duration of disease, Unified Parkinson’s Disease Rating Scale (UPDRS) score, Hoehn and Yahr (H&Y) stage. NMS and quality of daily life were assessed using the Non-Motor Symptoms Scale (NMSS) and 39-item Parkinson’s disease Questionnaire (PDQ-39), respectively. The Hamilton Anxiety Depression Scale (HAMD) was used to assess depression severity; and (3) global cognitive assessment using the Montreal Cognitive Assessment (MoCA) scales.

A recent study on the global scale for cognitive screening in PD suggested that among the 12 cognitive scales, the MoCA scale was classified as the “recommended level,” meaning that it meets all required criteria (Skorvanek et al., 2018). The scale mainly covers the assessment of visuospatial/executive function, namely attention, abstraction, language, memory, and orientation. Following the recommendations of this review, based on the UK brain bank diagnostic criteria for primary PD and the symptoms of cognitive impairment, we defined a MoCA score of < 21 as the cutoff score for PDD (Skorvanek et al., 2018). All PD patient evaluations were assessed during the “on” state and performed by trained physicians.

### Blood pressure measurements

Blood pressure (BP) measurements were performed in a quiet, temperature-controlled room set at 22–24°C. All participants fasted for > 2 h post-prandial to minimize post-prandial hypotension. Besides, they should avoid strenuous exercise and caffeine and to stop taking drugs affecting the autonomic nerves for at least 12 h. BP measurement was carried out by asking the patient to rest in a supine position for > 10 min; thereafter, both systolic blood pressure (SBP) and diastolic blood pressure (DBP) measurements were recorded in a 10-min supine position and a 10-min standing position.

Orthostatic hypotension was defined as any of the following conditions: (1) initial OH, a transient reduction in SBP ≥ 40 mm and/or DBP ≥ 20 mmHg within 15 s of standing; (2) classical OH, decreases in SBP ≥ 20/DBP ≥ 10 mmHg within 3 min of standing; or (3) delayed OH, drops in BP ≥ 20/10 mmHg after 3 min of standing (Freeman et al., 2018). Based on the BP results, we divided the patients into PD patients without OH (PD-NOH) and PD patients with OH (PD-OH).

### Dynamic cerebral autoregulation measurement and analysis

Dynamic cerebral autoregulation assessments were carried out by specialized vascular ultrasound physicians in accordance with the recommendations of the International Cerebral Autoregulation Research Network (the updated name: Cerebrovascular Research Network, CARNet) (Claassen et al., 2016). Continuous cerebral blood flow velocity (CBFV) was measured in the bilateral middle cerebral artery at a depth of 50–65 mm through the temporal window with a 2-MHz probe using a transcranial Doppler sonography (TCD) (EMS-9D Pro; Delica Medical, Shenzhen, China), and non-invasive continuous beat-to-beat BP (NIBP) was recorded using a servo-controlled plethysmograph (Finometer, Enschede, Netherlands) attached to the finger. Before each NIBP measurement, brachial BP was measured using a sphygmomanometer (Omron HBP-1300; Kyoto, Japan) to calibrate the baseline BP signal. The sampling frequency of the Doppler trace and NIBP signal was 125 HZ. And end-tidal carbon dioxide (ET-CO2) was also monitored using a nasal cannula connected to the EMS-9D Pro. Continuous CBFV, NIBP and HR were simultaneously recorded of each patient in the resting supine state for 10 min and in the active standing position for 10 min, respectively.

Based on the recommendation of CARNet, the stored and processed data were adopted using the transfer function analysis (TFA) method to reflect the oscillations in BP and cerebral blood flow at a range of frequencies. Based on the TFA method, we select the stable 5-min beat-to-beat BP and TCD monitoring raw data from the recorded 10 min of each posture for dCA analysis. And the length of the Hanning window was 100 s, and the superposition was 50%. We calculated the bilateral hemisphere phase, normalized gain (%/mmHg), absolute gain (cm/s/mmHg), and coherence at a very low frequency (VLF, 0.02–0.07 Hz) and low frequency (LF, 0.07–0.20 Hz) separately. The average value of dCA parameters in the bilateral hemispheres was used for further analysis. In general, the gain and phase reflect the amplitude and temporal relationship between BP (input signals) and CBFV (output signals) at the same frequency, whereas coherence approaches 1.0, reflecting the linear relationship between oscillations in BP and CBFV. Thus, a higher gain and lower phase shift represent impaired dCA; in addition, the phase is more reliable than other TFA parameters. Previous study has shown a low coherence (< 0.5) indicates that the linearity condition relating changes in velocity to pressure may be violated in this frequency range (Zhang et al., 1998). So in this study, only the autoregulatory parameters were used if the coherence was within ≥ 0.50 for further statistical analysis.

### Statistical analysis

All statistical tests were performed using IBM SPSS statistical software package (version 22.0; SPSS, IBM, New York, United States). Normally distributed continuous variables are expressed as the mean ± standard deviation (SD) and compared using *t*-tests, whereas skewed continuous variables are expressed as the median with interquartile range (IQR) and compared using the Mann–Whitney U test. Categorical variables, such as sex and risk factors (i.e., history of hypertension), are expressed as percentages and analyzed using Chi-square tests and a Fisher’s exact test. dCA parameters were compared between the PD-OH, PD-NOH and controls by using the *ANOVA* test, with Bonferroni correction for *post hoc* analysis if the overall group was statistically different. Comparison of group differences in coherence between supine and standing positions using paired *t*-test. Logistic regression models were used to identify the clinical factors associated with PDD and estimate their odds ratios (ORs) and 95% confidence intervals (CIs). All statistical tests performed were two-sided, and statistical significance was set to *p* < 0.05.

## Results

### Demographic information of participants

A total of 109 patients were enrolled in this study, including 89 patients with PD and 20 age- and sex-matched HC individuals. There were no significant differences between the two groups in terms of age, sex, SBP, DBP, or HR. The basic demographic data of patients are presented in Table 1.

**TABLE 1:** Demographic characteristics of participants.

	PD (*n* = 89)	HC (*n* = 20)	*P*
Age (years)	59.85 ± 10.54	59.20 ± 10.34	0.802
Male (*n*, %)	61 (68.5)	13 (65.0)	0.759
SBP (mmHg)	122.02 ± 16.89	126.83 ± 14.18	0.240
DBP (mmHg)	68.91 ± 12.23	77.61 ± 11.33	0.004*
HR (beats/min)	73.57 ± 11.44	70.31 ± 8.74	0.235

PD, Parkinson’s disease; HC, healthy control; SBP, systolic blood pressure; DBP, diastolic blood pressure; HR, heart rate.

*P < 0.05.

### Clinical characteristics of Parkinson’s disease patients

Among the 89 patients with PD, 26 (29.2%) included those with PDD who had a more advanced age, lower educational level, lower MoCA cognitive scale scores, and a significantly higher prevalence of OH than those without dementia (all *P* < 0.05); PDD was also associated with higher UPDRS scores. Of all patients with PD, 21 (23.6%) presented with OH, with initial OH was 52.4%, classic OH was 33.3% and delayed OH was 14.3%. However, sex, BMI, hypertension, disease duration, H&Y stage, and other PD-related scale scores, including UPDRS part III, NMSS, PDQ-39, and HAMD, did not significantly differ between patients with and without PDD. The detailed demographic and clinical data of the patients are presented in Table 2.

**TABLE 2:** Detailed demographic and clinical information of PD patients.

	PD (*n* = 89)	PD-N (*n* = 63)	PDD (*n* = 26)	*P*
Age, years	59.85 ± 10.54	58.19 ± 10.70	63.88 ± 9.14	0.020
Male (*n*, %)	61 (68.5)	42 (66.7)	19 (73.1)	0.554
Education (*n*, %)				< 0.001
Junior high school and below	38 (42.7)	19 (30.2)	19 (73.1)	
Senior high school	30 (33.7)	24 (38.1)	6 (23.1)	
College and above	21 (23.6)	20 (31.7)	1 (3.8)	
BMI (kg/m^2^)	24.42 ± 3.40	24.25 ± 3.41	24.82 ± 3.40	0.483
Hypertension (*n*, %)	24 (27.0)	15 (23.8)	9 (34.6)	0.296
Tremor	42 (47.2)	31 (49.2)	11 (42.3)	0.553
Bradykinesia	79 (88.8)	53 (87.3)	24 (92.3)	0.717
Rigidity	79 (89.8)	58 (93.5)	21 (80.8)	0.117
RBD	37 (41.6)	29 (46.0)	8 (30.8)	0.184
Disease duration, years	3.0 (1.5–5.0)	2.0 (1.5–5.0)	3.25 (2.0–5.25)	0.239
H&Y stage	2.20 ± 0.79	2.10 ± 0.71	2.44 ± 0.95	0.068
UPDRS	45.0 (30.0–75.5)	41.0 (29.0–62.5)	68.5 (38.0–78.75)	0.034
UPDRS part III	30.51 ± 15.95	28.42 ± 15.95	35.32 ± 14.29	0.065
NMSS	27.0 (11.0–47.25)	22.0 (9.75, 44.25)	37.5 (17.75–59.0)	0.144
PDQ-39	23.0 (14.0–46.0)	21.5 (14.0–41.5)	30.0 (11.0–58.0)	0.212
HAMD	6.0 (3.0–11.0)	6.0 (3.0–10.0)	5.0 (3.25–13.25)	0.886
MoCA	23.11 ± 4.27	25.24 ± 2.63	17.96 ± 2.85	< 0.001
OH (*n*, %)	21 (23.6)	10 (15.9)	11 (42.3)	0.008
OH subtypes (*n*, %)				
Initial OH	11 (12.3)	4 (6.3)	7 (26.9)	
Classic OH	7 (7.9)	5 (7.9)	2 (7.7)	
Delayed OH	3 (3.4)	1 (1.6)	2 (7.7)	

Values are expressed as mean ± SD, median (IQR) or *n* (%). PD-N, patients with Parkinson’s disease without dementia; PDD, patients with Parkinson’s disease with dementia; BMI, body mass index; RBD, rapid eye movement sleep behavior disorder; H&Y, Hoehn and Yahr Scale; UPDRS, unified Parkinson’s disease rating scale; NMSS, non-Motor Symptoms Scale; PDQ-39, 39-item Parkinson’s disease Questionnaire; HAMD, Hamilton Depression Rating Scale; MoCA, Montreal Cognitive Assessment; OH, orthostatic hypotension.

### Cognitive domains in Montreal Cognitive Assessment of patients with and without orthostatic hypotension

As shown in Table 3, The MoCA subdomain, and including visuospatial/executive function, attention, language and orientation scores of patients with OH were lower than those of non-OH patients. In particular, there were significant differences in the orientation and language cognitive domains, the latter consisting mainly of verbal repetition and fluency.

**TABLE 3:** Cognitive domains in MoCA of the PD patients with and without OH.

	PD-OH (*n* = 21)	PD-NOH (*n* = 68)	*P*
Visuospatial/executive	3.0 (2.0–5.0)	4.0 (3.0–5.0)	0.165
Naming	3.0 (2.0–3.0)	3.0 (2.0–3.0)	0.969
Attention	5.21 ± 1.08	5.44 ± 1.03	0.145
Language	1.0 (1.0–3.0)	2.0 (2.0–3.0)	0.027
Abstraction	2.0 (0–2.0)	2.0 (1.0–2.0)	0.525
Delayed Recall	2.0 (0–3.0)	2.0 (1.0–4.0)	0.472
Orientation	5.0 (5.0–6.0)	6.0 (6.0–6.0)	0.012

Values are expressed as mean ± SD, median (IQR). PD-OH, PD patients with orthostatic hypotension; PD-NOH, PD patients without orthostatic hypotension.

### Dynamic cerebral autoregulation parameters in Parkinson’s disease patients with or without orthostatic hypotension and healthy control

**FIGURE 1:**
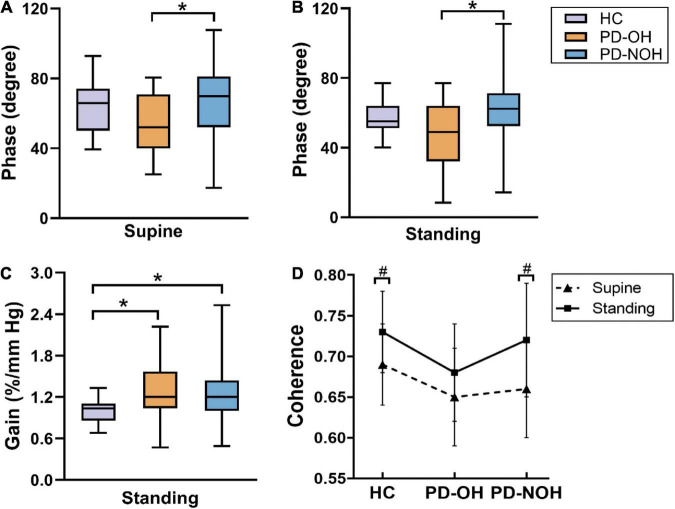
Comparison of autoregulation parameters between groups in VLF. **(A,B)** The phase difference from the PD-OH group is significantly lower than that of PD-NOH on both body postures. **(C)** During standing, there is significant difference in normalized gain between three groups. **(D)** The coherence of the standing position is higher than that of the supine in PD-NOH and HC. *Indicates *P* < 0.05; **^#^**Indicates *P* < 0.05 for comparison with supine; The whiskers in subplots **(A–C)** indicate the data range, and the error bars in subplot **(D)** indicates standard deviation. Eighty-nine PD patients and 20 HC in supine, 83 PD patients, and 20 HC in standing.

Table 4 shows the dCA indices at the VLF and LF range between the different groups in either the supine or standing posture. Six patients with PD were excluded from the analysis because they had difficulty completing the 10-min orthostatic challenge, resulting in unsatisfactory dCA monitoring data. Therefore, the final standing posture analysis included 83 patients with PD. In the VLF, the phase value in the patients with PD-OH was lower than that in those without OH on both body postures (supine: *P* = 0.042; standing: *P* = 0.029). During standing, the phase of PD-OH at LF is also significantly lower than that of HC group. Additionally, both in the VLF and LF range, the normalized gain of the orthostatic challenge differed significantly among the three groups (normalized gain at VLF: *P* = 0.011; normalized gain at LF: *P* < 0.001, respectively). Lower phase difference and higher gain present impairment of dCA function. There were no significant differences in the absolute gain in each group in the two-body posture (*P* > 0.05), and coherence in HC and PD-NOH groups when standing was higher than that in supine posture in VLF. The dCA parameters for each group in VLF are presented in Figure 1. Besides, BP and CBFV in each group during supine and standing positions was also had provided in Supplementary Table 1. In the standing position, mean arterial blood pressure and DBP in the PD-OH group were significantly lower than those in the non-OH and HC (*P* = 0.001).

**TABLE 4:** Comparison of cerebral autoregulation parameters in each group.

	VLF	PD-OH	PD-NOH	HC (*n* = 20)	*P*
Supine	Gain [cm/(s⋅mm Hg)]	0.77 ± 0.36	0.73 ± 0.27	0.69 ± 0.22	0.641
	Gain (%/mm Hg)	1.35 ± 0.49	1.30 ± 0.38	1.14 ± 0.43	0.219
	Phase (degree)	55.21 ± 17.98^†^	67.38 ± 21.12	64.05 ± 14.33	0.048
	Coherence	0.65 ± 0.06	0.66 ± 0.06	0.69 ± 0.05	0.136
Standing	Gain [cm/(s⋅mm Hg)]	0.62 ± 0.30	0.61 ± 0.22	0.60 ± 0.16	0.939
	Gain (%/mm Hg)	1.32 ± 0.44*	1.24 ± 0.37*	1.00 ± 0.18	0.011
	Phase (degree)	48.50 ± 18.06^†^	59.43 ± 16.98	57.18 ± 9.82	0.034
	Coherence	0.68 ± 0.06	0.72 ± 0.07^†^	0.73 ± 0.05^†^	0.012

	**LF**				

Supine	Gain [cm/(s⋅mm Hg)]	0.95 ± 0.35	0.97 ± 0.46	0.89 ± 0.23	0.742
	Gain (%/mm Hg)	1.71 ± 0.52	1.60 ± 0.50	1.47 ± 0.28	0.269
	Phase (degree)	45.35 ± 17.03	47.04 ± 14.71	45.25 ± 9.10	0.847
	Coherence	0.68 ± 0.09*	0.70 ± 0.08*	0.77 ± 0.06	0.002
Standing	Gain [cm/(s⋅mm Hg)]	0.93 ± 0.43	0.87 ± 0.31	0.77 ± 0.20	0.264
	Gain (%/mm Hg)	2.04 ± 0.59*^†^	1.76 ± 0.44*	1.28 ± 0.23	0.001
	Phase (degree)	35.62 ± 12.15*	38.98 ± 9.70	45.11 ± 11.95	0.020
	Coherence	0.70 ± 0.08	0.75 ± 0.08	0.75 ± 0.05	0.052

VLF, very low frequency; LF, low frequency; PD-OH, PD patients with orthostatic hypotension; PD-NOH, PD patients without orthostatic hypotension; HC, healthy control. 89 patients in supine (PD-OH = 21, PD-NOH = 68), 83 patients in standing (PD-OH = 20, PD-NOH = 63).

*P < 0.05 for comparison with HC, ^†^*P* < 0.05 for comparison with PD patients without OH. *ANOVA* test was used to compare dCA parameters in each group, and *post hoc* analysis was corrected by Bonferroni.

### Dynamic cerebral autoregulation parameters in Parkinson’s disease patients with or without dementia

Comparison of cerebral autoregulation parameters in the PD patients with and without dementia are presented in Table 5. During standing posture, the LF normalized gain of the patients with PDD was significantly higher than that of the PD patients without dementia (*P* = 0.015), which indicates that the cerebral autoregulation function of PDD patients is impaired. Low-frequency dCA parameters in the supine position were not observed to differ between groups in PD patients with dementia and non-dementia.

**TABLE 5:** Comparison of cerebral autoregulation parameters in the PD patients with and without dementia.

	VLF	PD-N	PDD	*P*
Supine	Gain [cm/(s⋅mm Hg)]	0.74 ± 0.29	0.75 ± 0.29	0.891
	Gain (%/mm Hg)	1.30 ± 0.40	1.33 ± 0.43	0.788
	Phase (degree)	64.00 ± 21.79	65.75 ± 19.32	0.722
	Coherence	0.66 ± 0.07	0.65 ± 0.06	0.499
Standing	Gain [cm/(s⋅mm Hg)]	0.63 ± 0.25	0.57 ± 0.22	0.259
	Gain (%/mm Hg)	1.26 ± 0.41	1.23 ± 0.34	0.745
	Phase (degree)	55.52 ± 18.00	59.75 ± 17.19	0.322
	Coherence	0.72 ± 0.07	0.71 ± 0.08	0.625

	**LF**			

Supine	Gain [cm/(s⋅mm Hg)]	0.95 ± 0.37	0.96 ± 0.39	0.942
	Gain (%/mm Hg)	1.56 ± 0.44	1.78 ± 0.62	0.074
	Phase (degree)	47.65 ± 14.20	44.15 ± 17.49	0.333
	Coherence	0.70 ± 0.09	0.68 ± 0.09	0.211
Standing	Gain [cm/(s⋅mm Hg)]	0.87 ± 0.30	0.92 ± 0.42	0.586
	Gain (%/mm Hg)	1.74 ± 0.42	2.03 ± 0.58	0.015
	Phase (degree)	38.09 ± 9.44	38.57 ± 12.45	0.848
	Coherence	0.71 ± 0.08	0.75 ± 0.09	0.02

VLF, very low frequency; LF, low frequency; PD-N, patients with Parkinson’s disease without dementia; PDD, patients with Parkinson’s disease with dementia. Eighty-nine patients in supine (PD-N = 63, PDD = 26), 83 patients in standing (PD-N = 59, PDD = 24). *T-tests* was used to compare dCA parameters in each group.

### Risk factors associated with Parkinson’s disease dementia

Univariate logistic regression analysis revealed age, education level, OH and LF normalized gain in standing were significant predictors of PDD (*P* < 0.05). In the adjusted model, the results of the multivariate logistic regression analyses showed that educational level and LF normalized gain in standing are significantly related factors for PDD. In other words, patients with a higher level of education were at lower risk for developing dementia, and a higher LF normalized gain in standing (impaired dCA) was positively associated with dementia in PD patients (Table 6).

**TABLE 6:** Univariate and multivariate logistic regression analysis of influencing factors for PDD.

	Variables	OR	95% CI	*P*
Univariate	Age	1.059	1.007–1.113	0.024
	Education			
	Junior high school and below	Reference		
	Senior middle school	0.250	0.083–0.749	0.013
	College and above	0.059	0.006–0.411	0.005
	Disease duration	0.988	0.869–1.124	0.857
	H&Y stage	1.730	0.949–3.153	0.074
	UPDRS total	1.019	0.999–1.038	0.061
	UPDRS part III	1.028	0.998–1.060	0.070
	OH	3.887	1.387-10.892	0.010
	LF Gain (%/mm Hg) (in standing)	3.292	1.204–9.002	0.020
Multivariate	Education			
	Junior high school and below	Reference		
	Senior middle school	0.231	0.067–0.795	0.020
	College and above	0.072	0.008–0.634	0.018
	LF Gain (%/mm Hg) (in standing)	3.756	1.241–11.367	0.019

OR, odds ratio; CI, confidence interval. Variables with a *P* value of < 0.1 in univariate analysis were eligible for inclusion in the multiple logistic regression model.

## Discussion

In this study, we explored the prevalence and association of OH and dementia in patients with PD. Patients with OH exhibited poorer performance in the cognitive domains of language and orientation compared with those without OH. Although OH and cognitive deficits are common NMS in patients with PD, only a few studies have investigated the underlying mechanisms between them. To our knowledge, this is the first study reveal that impaired dCA is a potential mechanism of OH and cognitive impairment based on the assessment of cerebral autoregulation in patients with PD from the supine to standing position.

We found that the prevalence of OH was 23.6%, similar to the results of a meta-analysis that more precisely estimated the pooled rates of OH in PD as 30.1% (95% CI: 22.9–38.4%) (Velseboer et al., 2011). Although the relationship between OH and cognitive impairment in PD remains controversial and the mechanisms of their association remain unclear, increasing evidence points to a positive correlation between OH and cognitive impairment, particularly regarding executive dysfunction (usually related to frontal lobe dysfunction), which is the most common domain-specific affected of PD (Kim et al., 2012; Pilleri et al., 2013; Udow et al., 2016; Longardner et al., 2020). In this study, PD patients with OH scored relatively lower in visuospatial/executive function than those without OH. Interestingly, a review reported that verbal fluency (such as asking patients to name as many animals as possible within 1 min) is also a part of the executive syndrome (Gratwicke et al., 2015). According to a cohort study of patients with *de novo* PD, Bae et al. (2014) demonstrated that OH was clearly related to the verbal memory cognitive domain, which is consistent with our findings showing that individuals with OH had worse performance on tests of verbal repetition and fluency and orientation function.

Considering the strong association between OH and cognitive dysfunction in PD, several systematic reviews have provided a detailed explanation of the pathological mechanisms underlying this relationship. First, synaptic proteins are deposited in the peripheral ANS, followed by a caudoraustral pattern ascending from the brainstem to the cortex, resulting in progressive dopaminergic neuronal and noradrenergic degeneration. Therefore, the ANS alterations may appear early in PD owing to brainstem involvement, even prior to the onset of cognitive deficits and presentation of typical motor symptoms. Alternatively, recurrent hypotension may cause cerebral blood flow (CBF) fluctuations and hypoperfusion, leading to subcortical vascular damage (McDonald et al., 2016). In brief, the interaction of the above pathophysiological mechanisms may contribute to OH and cognitive impairment and progressively exacerbate disease progression and severity (McDonald et al., 2016; Udow et al., 2016).

As a cerebrovascular mechanism, cerebral autoregulation ensures that CBF remains stable despite changes in BP (van Beek et al., 2008). An earlier study demonstrated that disrupted cerebrovascular autoregulation usually precedes the onset of cognitive deficits, illustrating its critical role in the mechanism of dementia progression (Girouard and Iadecola, 2006). Vokatch et al. (2007) and Tsai et al. (2009) successively provided evidence of impaired cerebral autoregulation in PD patients; however, they did not assess the condition of dCA in OH. In this study, we compared the dCA differences not only in the resting position but also in the active-standing maneuver. The autoregulatory parameters, both in the supine and standing positions, were compromised in patients with OH compared to those without OH. Meanwhile, the normalized gain at VLF and LF range was markedly higher in the OH and non-OH groups when standing than in the HC group. These findings indicated that dCA function was impaired in patients with OH. Recently, post-stroke cognitive impairment (PSCI) patients were revealed to have distinctly worse phases of dCA indices than non-PSCI and HCs, and impaired dCA (VLF-phase ≤ 46°) was involved in the progression of PSCI (Chi et al., 2020). This conclusion is similar to our findings that PDD patients have worse dCA function than non-dementia. Interestingly, an apparently increased coherence in PD-NOH and HC group during an orthostatic challenge as compared to a supine position was observed in our study. This higher coherence is usually indicative of the reliability of the assessed dCA indices, which is consistent with the results of the latest research performed by Favre et al. (2020) in healthy individuals.

Parkinson’s disease dementia has increasingly been accepted as the final stage of cognitive impairment, with a cumulative prevalence of 75–90% for a disease duration of ≥ 10 years. In addition, a recent meta-analysis suggested that PD duration (>10 years) was an important factor in increasing the frequency of PDD (Severiano et al., 2022). However, the duration of PD was not observed to be a significant factor of PDD in this study, which may require further exploration with cohort studies. PDD development negatively affects one’s quality of life on a daily basis and results in strikingly increased caregiver burden and even mortality (Gratwicke et al., 2015). A systematic review of 36 studies showed that the prevalence of dementia in PD is 24–31% (Aarsland et al., 2005). Our results are consistent with these findings, showing a rate of 29.2%.

A strength of this study was our analysis of the clinical factors associated with PDD. A prospective study by Hussain and Camicioli (2018) confirmed that only advanced age and OH in patients with PD are correlated with an increased risk of developing dementia. Our results also showed that age and OH in patients with PD was relevant to dementia in univariate analysis, and McDonald et al. demonstrated that recurrent episodes of OH can lead to cerebral hypoperfusion, in turn contributing to impaired cognitive function (McDonald et al., 2016). In our final multivariate model, higher LF normalized gain in standing (i.e., impaired dCA) was associated with an increased risk of PDD, similar to the results of a supine-to-standing TCD study that showed impaired cerebral autoregulation and decreased efficiency of CBF regulation in Alzheimer’s disease (Zhou et al., 2019). In a related long-term cohort study, cerebral hypoperfusion was correlated with accelerated cognitive impairment and increased risk of dementia in the general population (Wolters et al., 2017), and that intact CA function is a fundamental regulatory mechanism that protects cerebral perfusion from changes in BP. In the current study, PD patients with OH also have worse dCA function. Taken together, these findings indicate that dCA damage may be the underlying mechanism of OH and dementia in patients with PD. Furthermore, the results of a prospective population-based study, i.e., individuals with PD and OH had lower Mini-Mental State Examination (MMSE) cognitive scores over a 7-year follow-up period (Hiorth et al., 2019), which suggesting OH is related to cognitive deficits over time, and OH in PD should be more actively assessed and managed in the clinic. Additionally, we found that higher education might exert a protective effect on cognitive decline in PD; a previous comprehensive review shed light on the similar points, demonstrating that less educated individuals are at increased risk of developing PDD, consistent with the cognitive reserve hypothesis (Poletti et al., 2011).

This study had some limitations. First, the exclusion of six patients who were unable to stand for 10 min may have caused a bias in the analysis of standing dCA data, thereby potentially leading to an overestimation of OH in the relationship between dCA and cognition. Second, we only used the MoCA scale to assess the cognitive impairment in PD, and our future research needs to combine other cognitive scales for comprehensive neuropsychological testing. Besides, neurogenic OH and supine hypertension is a common manifestation of cardiovascular dysautonomia and coexists with hemodynamic abnormalities in PD, has been considered as negative prognostic factors for cardio- and cerebrovascular events and cognitive impairment (Espay et al., 2016; Fanciulli et al., 2016); However, our study did not determine whether there is a relationship between neurogenic OH or supine hypertension and dCA; this topic warrants further investigation. Lastly, considering the single-center cross-sectional design of this study, patients with cognitive impairment were not followed up; therefore, additional studies with longer term follow-up to observe changes in cognition relative to dCA are required to validate our findings.

Overall, our study demonstrated that there are differences in the dCA parameters of PD patients with and without OH, highlighting that impaired cerebral autoregulation may be a potential mediator of OH and cognitive impairment in patients with PD. Therefore, evaluating dCA in PD patients based on BP and cerebral hemodynamics in clinic is helpful for early diagnosis of OH and effective intervention may be expected to reduce the risk of cognitive impairment.

## Data availability statement

The raw data supporting the conclusions of this article will be made available by the authors, without undue reservation.

## Ethics statement

The studies involving human participants were reviewed and approved by the Ethics Committee of the Xuanwu Hospital, Capital Medical University. The patients/participants provided their written informed consent to participate in this study.

## Author contributions

HC and YX conceived and designed the study and interpreted the results of data. HC, YX, EX, FZ, QL, JZ, and SM contributed to the data collection and methodology. HC performed the statistical analyses and drafted the manuscript. YX edited and revised the manuscript. All authors read and approved the final manuscript.
