# Supplementary material for: Impaired dynamic cerebral autoregulation: A potential mechanism of orthostatic hypotension and dementia in Parkinson’s disease
Source: Front Aging Neurosci. 2022 Sep 8;14:927009. doi: 10.3389/fnagi.2022.927009 (PMC9492951; doi:10.3389/fnagi.2022.927009)
Supplement: Supplementary file 1 [file Table_1.DOCX]

**Supplementary Table 1. Blood pressure and cerebral blood flow velocity in each group**

|  |  | PD-OH | PD-NOH | HC (n=20) | *P* | |
| --- | --- | --- | --- | --- | --- | --- |
| Supine |  |  |  |  | |  |
|  | SBP (mmHg) | 127.88 ± 17.50 | 120.50± 13.40 | 123.42 ± 8.96 | | 0.103 |
|  | DBP (mmHg) | 69.52 ± 12.51 | 70.15 ± 11.07 | 75.13± 9.65 | | 0.221 |
|  | MAP (mmHg) | 86.36 ± 13.18 | 86.61± 11.01 | 89.59 ± 6.94 | | 0.603 |
|  | PSV (cm/s) | 86.07 ±23.65 | 85.56 ± 15.54 | 84.82 ± 21.95 | | 0.981 |
|  | EDV (cm/s) | 34.62 ± 11.27 | 37.65 ± 8.59 | 39.15 ± 12.62 | | 0.274 |
|  | MV (cm/s) | 51.96± 15.34 | 53.60 ± 10.40 | 54.19 ± 12.78 | | 0.819 |
|  | ET-CO_2_ | 38.23± 1.78 | 38.78 ± 2.82 | 38.89 ± 2.07 | | 0.873 |
| Standing |  |  |  |  | |  |
|  | SBP (mmHg) | 107.81 ± 27.10 | 120.18 ± 15.14 | 117.63 ± 18.82 | | 0.034 |
|  | DBP (mmHg) | 59.29 ± 18.91*^,^† | 75.20 ± 10.69 | 75.31 ± 10.35 | | 0.001 |
|  | MAP (mmHg) | 75.46 ± 19.47*^,^† | 89.95 ± 11.19 | 89.42 ± 10.70 | | 0.001 |
|  | PSV (cm/s) | 77.98 ± 21.31 | 79.68 ± 15.65 | 82.34 ± 11.71 | | 0.756 |
|  | EDV (cm/s) | 30.26 ± 7.97 | 33.79 ± 8.96 | 36.18 ± 10.54 | | 0.159 |
|  | MV (cm/s) | 45.23 ± 12.05 | 49.08 ± 10.40 | 52.18 ± 11.53 | | 0.167 |
|  | ET-CO_2_ | 38.63± 1.68 | 38.97 ± 2.4 | 38.41 ± 2.65 | | 0.874 |

PD-OH, PD patients with orthostatic hypotension; PD-NOH, PD patients without orthostatic hypotension; HC, healthy control; SBP, systolic blood pressure; DBP, diastolic blood pressure; MAP, mean blood pressure;; PSV, peak systolic velocity; EDV, end diastolic velocity; MV, mean velocity; Et-CO2, end-tidal carbon dioxide

* P<0.05 for comparison with HC, † P<0.05 for comparison with PD patients without OH.

*ANOVA* test was used to compare dCA parameters in each group, and post-hoc analysis was corrected by Bonferroni.
